# Publisher Correction: Integrated analysis of a compendium of RNA-Seq datasets for splicing factors

**DOI:** 10.1038/s41597-020-00607-x

**Published:** 2020-08-07

**Authors:** Peng Yu, Jin Li, Su-Ping Deng, Feiran Zhang, Petar N. Grozdanov, Eunice W. M. Chin, Sheree D. Martin, Laurent Vergnes, M. Saharul Islam, Deqiang Sun, Janine M. LaSalle, Sean L. McGee, Eyleen Goh, Clinton C. MacDonald, Peng Jin

**Affiliations:** 1 West China Biomedical Big Data Center, West China Hospital, Sichuan University, Chengdu, China; 2 Medical Big Data Center, Sichuan University, Chengdu, China; 3 Center for Epigenetics & Disease Prevention, Institute of Biosciences and Technology, College of Medicine, Texas A&M University, Houston, TX 77030 USA; 4 School of Electronic and Information Engineering, Suzhou University of Science and Technology, Suzhou, Jiangsu 215009 China; 5 Department of Human Genetics, Emory University School of Medicine, Atlanta, Georgia USA; 6 Department of Cell Biology & Biochemistry, Texas Tech University Health Sciences Center, Lubbock, Texas 79430 USA; 7 Neuroscience Academic Clinical Programme, Duke-NUS Medical School, NA, Singapore; 8 Metabolic Reprogramming Laboratory, Metabolic Research Unit, School of Medicine and Centre for Molecular and Medical Research, Deakin University, Geelong, Victoria Australia; 9 Department of Human Genetics, David Geffen School of Medicine, University of California-Los Angeles, Los Angeles, CA USA; 10 Department of Medical Microbiology and Immunology, Genome Center, and MIND Institute, University of California Davis, Davis, CA USA

Correction to: *Scientific Data*10.1038/s41597-020-0514-7, published online 16 June 2020

After publication it was noted that Tables S1–S10 and Data S1-S3 which are available at figshare were incorrectly linked to the Supplementary File. The links have been removed from the HTML version of this paper and the correct figshare URLs have been added to the text in both the HTML and PDF versions of this manuscript.

